# Toy Preferences among 3-to-4-Year-Old Children: The Impact of Socio-Demographic Factors and Developmental Characteristics

**DOI:** 10.11621/pir.2023.0206

**Published:** 2023-06-30

**Authors:** Margarita N. Gavrilova, Vera L. Sukhikh, Nikolay N. Veresov

**Affiliations:** a Lomonosov Moscow State University, Moscow, Russia; b Monash University, Melbourne, VIC, Australia

**Keywords:** child psychology, cultural-historical approach, play, toy preference, executive functions, emotion understanding

## Abstract

**Background:**

Today’s common typologies and categories of children's toys are mainly decided by the manufacturers and retailers of children's products. Such categorizations are not based on a theoretical understanding of child development and therefore cannot provide information about the opportunities that toys provide for the young.

**Objective:**

This study proposed three criteria for categorizing toys based on the cultural-historical approach: their degree of realism; their degree of anthropomorphism; and their degree of detail. These criteria were chosen as a result of an analysis of theoretical works carried out in the framework of cultural-historical approach.

**Design:**

The proposed criteria were tested through an experiment measuring children's toy preferences. The participants were 129 children of ages 3-4 years. Experimental data confirmed that most children do prefer realistic and detailed toys rather than those with fewer of these properties. The contribution of socio-demographic factors and the children's individual developmental indicators to their toy preference was also analyzed.

**Results:**

The study revealed that among various socio-demographic factors, only the child’s gender and the number of siblings in the family acted as significant predictors for the toy preferences. None of child’s developmental characteristics (nonverbal intelligence, executive functions, and emotional understanding) were found to be significant predictors of preference for particular toys.

**Conclusions:**

The assumption that toys can be assessed in terms of their realism and degree of detail found empirical support. The results of this study may be useful in designing further research and in the practical issue of toy selection for children age 3-4 years.

## Introduction

A toy is not just an object; it is the material for children's play and a means of child development (Francis, 2010; Wynberg et al., 2022). Pre-school children are naturally motivated to play (Bondi et al., 2021; Guirguis, 2018; Lillard, 2017; Ryabkova et al., 2019; Veraksa et al., 2020; Whitebread, 2017, 2018). Previous studies have reported that play promotes the development of executive function skills (Doebel et al., Fleer et al., 2019; Kelly et al., 2011; Veraksa et al., 2020; Vidal Carulla et al., 2021); emotional and social development (Colliver et al., 2021; Howard et al., 2017; Mathieson et al., 2011); and speech (Nicolopoulou et al., 2015; Quinn et al., 2018).

Certain studies of child development and play have dealt with some aspects of the specifics of toys and their choice (Davis et al., 2020; Francis, 2010; Mertala et al., 2016; Wynberg et al., 2022; Fleer, 2022). Mostly, such works were focused on children's gender and age as the main factors determining their preferences (Ban et al., 2022; Hassett et al., 2008; Liu et al., 2020; Mertala et al., 2016). Only very few studies were in fact related to the role of individual psychological predictors of toy preferences (Francis, 2010; Liu et al., 2020). Some of them were conducted within the framework of the cultural-historical approach. For instance, a significant study under the guidance of Smirnova was done to create a whole methodology for determining the psychological and pedagogical expertise of skills toys can provide (Smirnova et al., 2008; Smirnova, 2011a; Smirnova, 2011b).

The relevance of conducting further research on toys and children's preferences is that, with the huge variety of contemporary toys, there is a lack of the evidence-based data needed to examine toys and their potential impact on child development. This often results in children being exposed to toys that not only do not contribute to their mental development, but can also be harmful (Smirnova et al., 2016).

### Toy categorization approaches

There is no single categorization of toys, since that process is based on different theoretical approaches and concepts (Kudrowitz & Wallace, 2010). The most common method of categorization is used for mass market toys. It is based on the particular physical category used in their production, like the materials and technology *(e.g.,* soft toys, dolls, bricks) (Clark, 2007). Another widespread method of toy categorization is based on the definition of the toys main function or expected developmental effect *(e.g.,* sensory toys, musical toys, puzzles) (Kudrowitz & Wallace, 2010). Such approaches, in addition to dividing toys by children's gender or age, are not sufficiently theoretically grounded. They can rather be seen as an attempt to structure the abundant toy market.

What is much more useful is to evaluate toys according to the opportunities they provide for a child to develop through play. Such an approach requires not only a detailed theoretical understanding of child development stages and mechanisms, but also extensive qualitative experience in observing children's play (Veraksa et al., 2022). Such a categorization of toys associated with Piaget's stages of cognitive development is described in the Kudrowitz & Wallace study (2010). Based on a review of hundreds of toys, the authors proposed four categories that define the “play value” of a toy: sensory, fantasy, construction, and challenge. They emphasized that each category of toys is of interest to children especially during the corresponding cognitive development periods (the sensory-motor period, preoperational stage, period of concrete operations, and period of formal operations).

From a cultural-historical point of view, the child recreates life experiences when playing. In this approach, play is considered one of the most important sources of development during the preschool years. The child not only acts out stories, but also learns about the nature of social relationships. Therefore, toys should have certain properties to help the child create an imaginary situation. Toy characteristics that should be of interest to children, according to the cultural-historical approach, are described below.

#### Realistic toys

Vygotsky emphasized that a toy should contribute to the creation of an imaginary or pretended situation (Vygotsky, 1972). Realistic toys are objects that are a small replica of real objects used by adults. They include, for example, home furnishings (furniture, dishes), food (fruit, vegetables), and themed sets (doctor, fireman, supermarket). Such toys encourage the child to play out familiar scenes.

#### Anthropomorphic toys

Elkonin has shown that most often children act out relationships between people in play (Elkonin, 2005). In other words, the main content of play is human interaction in different situations. Therefore, children should be more interested in toys that provide an opportunity to represent stories related to social interaction and human activities. Anthropomorphic toys may include those toys that have human features (family doll set, human figures).

#### Detailed toys

Smirnova emphasizes that the toy should have enough details and necessary attributes for recreating real situations in play (Smirnova, 2011). In this case the child will better understand what actions can be taken with the toy. A detailed toy will be of more interest to the child than the others, because it contains ‘Tints” as to what activities can be done with it.

### Main research paradigms on children's toy preferences

Children's preferences in toys are mostly measured by questionnaires or observation. Both children and the adults who spend a lot of time with them can become the respondents for the surveys (Sung, 2018). Some researchers opt for the format of a retrospective report, when adults report which toys they preferred in their young years. However, when a grown-up is interviewed, there is a risk of distorted information about his/her real preferences as a child. Moreover, the toy market changes over time. The observation of the process of choosing a toy can take place both in natural and experimental conditions (Hassett et al., 2008; Liu et al., 2020). The paradigms that define the way these observations are conducted can be divided into four categories: free play (Fagot et al. 1969; Pasterski et al., 2005); the naturalistic approach (Downs, 1983; Nelson, 2005); visual preference; or choosing among given options (Golombok, 2010).

### Current study

This study aimed to specify scientific data on young children's toy preferences. The research design was developed on the basis of the paradigm of a forced choice from a number of options. This approach ensures equal experimental conditions for data collection. The novelty of this study derives from its exploration of not only sociodemographic predictors, which have been studied previously (Davis & Hines, 2020), but also the chief developmental characteristics which may impact children's toy preferences. Among socio-demographic factors considered in this study were the gender and age of the children, the level of mother’s education, and the number of siblings cohabiting with the child. Non-verbal intelligence, executive functions, and emotional understanding were considered the indicators of the main developmental areas which are potentially able to impact toy preferences.

This study aimed to address the following research questions: a) Would the children prefer more realistic toys over those that are less related to children's real-life experiences? b) Would the children prefer more anthropomorphic toys to those with less human traits? c) Would the children prefer more detailed toys to those with few details and attributes? d) Do socio-demographic factors or developmental characteristics significantly predict a child’s preference in toys?

## Methodology

### Sample

One hundred twenty-nine 3-to-4-year-old children attending Moscow preschool institutions and their mothers participated in the study. Their average age was 42 months (3.92 y.o.). The proportion of male and female respondents was approximately equal (51% were girls). The level of education of the mothers who took the survey was distributed as follows: secondary vocational education = 4.9%; higher professional education (bachelor, master, or specialist) = 87%; and scientific degree = 4.8%. A few (2.4%) mothers refused to provide this information.

This data was collected from October to December 2022, and the procedure consisted of three stages. In the first, a parental survey was carried out to clarify the socio-demographic situation of the family. The survey was conducted by means of printed questionnaires that were handed out to the parents in the kindergarten. Only those children whose parents provided their answers were included in the study.

The second stage consisted of a developmental outcome assessment; several individual sessions were held with each child. The diagnosis was performed by experienced testers who had psychological training. All techniques followed the same order, and each session didn’t last more than 15 minutes. Not all the children completed all the tests (some children refused to complete certain tasks, and if that was the case, the assessment was terminated).

The third stage included individual sessions where each child was asked to choose one toy among others in three experimental trials.

### Tools

Five tools successfully validated for a Russian sample were used to measure the children’s cognitive regulation: 1) the *Dimension Card Change Sorting* (Zelazo, 2006) for the level of cognitive flexibility; 2) the subtest *Memory for Designs* (Korkman et al., 2007) for visual working memory; 3) the *Inhibition* subtest of NEPSY-II (Korkman et al., 2007) for inhibitory control; 4) the *Sentences Repetition* subtest of NEPSY-II (Korkman et al., 2007) for the volume of auditory working memory; and 5) the *Statue* subtest of NEPSY-II (Korkman et al., 2007) for the level of physical inhibitory control.

The Russian version of the *Test of Emotion Comprehension* (TEC) (Pons et al., 2000) was used to evaluate the children’s level of emotional development. The Russian version of the TEC has been successfully adapted and validated for use in a Russian sample (Veraksa et al., 2021). The test assesses three levels of emotional understanding: External, Mental, and Reflexive. The External level focuses on the child’s ability to recognize emotions, to understand the external causes of emotions, and the impact of desires on emotions. The Mental level concerns understanding the role of beliefs and memories on emotions, as well as understanding of hidden emotions. The Reflexive level is the most complex and evaluates understanding of mixed feelings, the possibilities of emotional regulation via cognitive strategies, and the influence of moral self-reflective rules on emotions.

Non-verbal fluid intelligence was assessed by means of the Russian adaptation of *Raven’s Coloured Progressive Matrices* (CMPM) (Raven et al., 2002).

A parental survey in the form of printed questionnaires was administered to collect the socio-demographic data (gender and age of the children, the level of the mother’s education, and the number of siblings living together with the child).

### Experimental procedure

**Figure 1.**
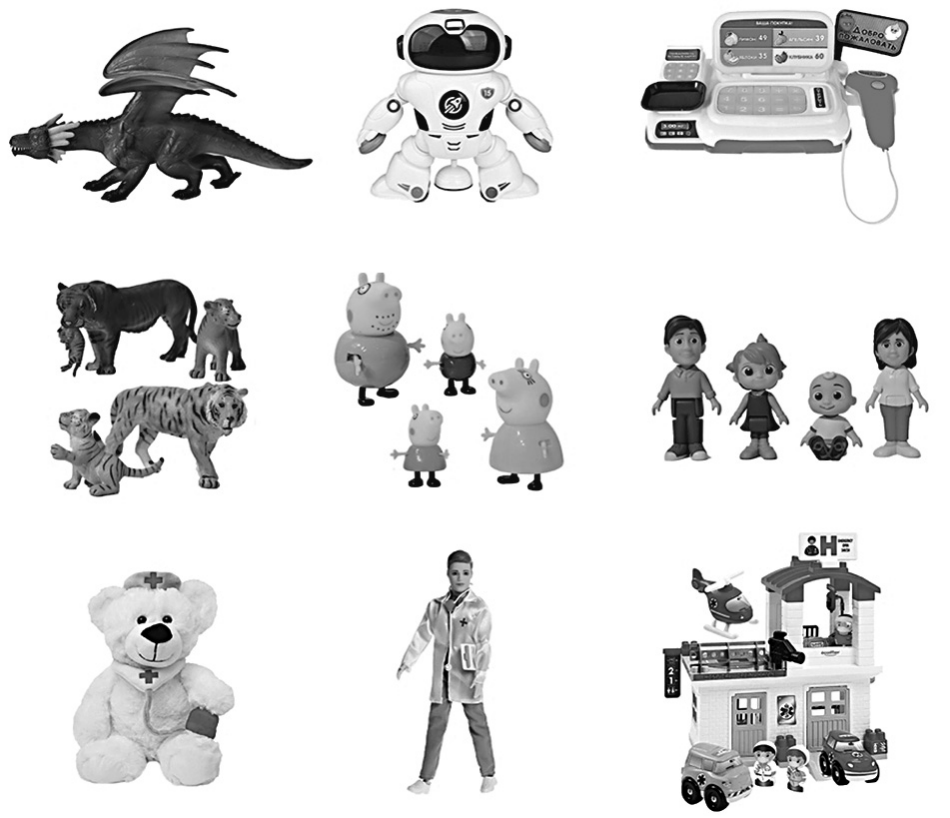
Toys presented in three toy-preference trials.

*Notes: a) toy realism trial with three levels (low=dragon, middle=space, high=shop); b) toy anthropomorphism trial with three levels (low=a tiger family, middle=a family of pigs, high=a human family); c) toy detail trial with three levels (low a doctor bear, middle=a doctor doll, high=a hospital play set)*.

The experimental session aimed at investigating the children’s toy preferences was held within the framework of a forced choice paradigm. The experiment included three trials corresponding to the research questions on toy preference. In each trial, the child was shown three toys, which had been selected based on the degree of the three variables (level of realism, anthropomorphism, or detail) (see *Figure* 1).

*The first trial* was designed to explore whether the children preferred more realistic toys to those that were less related to their real-life experiences. For this purpose, the three options were the following: “a) the shop where we go every day; b) the space where just a few people can travel; and c) the dragon that only exists in the fairy-tales.”

*The second trial* was designed to examine whether the children preferred more anthropomorphic toys to those with less human traits. In this trial the options to choose from were three sets of families: a human family, a family of pigs looking like people (Peppa Pig ™), and a tiger family.

*The third trial* was designed to investigate whether the children preferred more detailed toys to those with fewer details and attributes. The options were three toys relating to the same theme (playing doctor) but differing in degree of detail: a doctor bear, a doctor doll with medical paraphernalia, and a hospital play set.

In all the experimental sessions, the trials were carried out in an identical manner. The toys were equal in size and set out on a dense white cloth at the same distance from each other. While the experimenter established contact with the child and gave the instructions, the latter could not see the toys. Also, when one category of toys was presented, the others were out of sight. This instruction was given to the child: “Please take these two circles, a big and a small one (two cardboard circles were demonstrated). I will show you some toys now, and I will ask you to place the circles in such a way that they would show which toys you like more, and which less. The big circle should go with the toy you would like to play with the most, and the small circle with the one that you would pick in the last place.” The average total time of the experiment with each child was 9-10 minutes.

## Results

### Descriptive statistics

The results of individual testing of the participants’ intellectual, executive function, and emotional development are presented in *Table 1*. As a result of the examination, certain age-related specifics of mental development of 3-to-4-year-old children were revealed. The majority of children were unable to switch between rules while performing the cognitive flexibility tasks, and a lot of erroneous (impulsive) answers were given during the inhibitory control task, without any intention of correcting them. Moreover, the participants could only retain in their memory the words and the semantic elements of short and grammatically simple sentences. They also demonstrated low skills of understanding the reasons for their emotions.

**Table 1:** The results of individual testing of children's intellectual, executive function, and emotional development

	Range	*M*	*SD*	Min	Max
Nonverbal intelligence	0-36	5.73	3.78	1	21
Cognitive flexibility	0-24	16.36	2.30	8	23
Visual working memory	0-150	37.12	8.56	13	81
Inhibitory control (corrected errors)	0-40	1.65	1.85	0	8
Inhibitory control (uncorrected errors)	0-40	11.36	9.02	0	35
Auditory and verbal working memory	0-34	9.65	6.39	0	25
Understanding of external reasons of emotions	0-3	0.81	0.72	0	3
Understanding of mental reasons of emotions	0-3	0.86	0.73	0	3
Understanding of reflexive reasons of emotions	0-3	0.57	0.64	0	2
General emotional intelligence	0-9	2.25	1.35	0	7

*Notes. The “Inhibitory control (corrected errors)” variable refers to the number of errors that the child corrected while performing this task, while the “Inhibitory control (uncorrected errors)” variable refers to the number of errors that were left intact*.

However, if we study the results individually, the values for each factor varied. For instance, some children obtained almost the highest possible score in nonverbal intelligence, cognitive flexibility, and auditory and verbal working memory, already at the age of 3. They also managed to complete the inhibitory control task without any mistakes. The scores on visual working memory and understanding the reflexive reasons for emotions demonstrated the least dispersion in this sample.

*Table 2* provides the experimental data on toy preference among children. As can be seen from the table, more than half of the children (55.8%) preferred the most re-alistic toys over toys less connected to their real-life experiences. The least preferred toy in this trial was the dragon, which, according to the instructions, only exists in fairy tales (12.4%). In the anthropomorphism trial, the most popular choice was not a human family, as expected, but a family of pigs looking like people (42.6%). However, the most unpopular choice was the tiger family (25.6%). Finally, the majority of children (63.6%) preferred to choose more detailed toys among those with fewer details and attributes. The plush doctor-bear, which was the least detailed toy, was chosen by the children the least number of times (14.7%).

**Table 2:** Children's preferences on toy choice in the three experimental trials

Experimental trial	Options	Counts	% of Total
Degree of toy realism	Low level (dragon)	16	12.4 %
	Middle level (space)	41	31.8 %
	High level (shop)	72	55.8 %
Degree of toy anthropomorphism	Low level (a tiger family)	33	25.6 %
	Middle level (a family of pigs)	55	42.6 %
	High level (a human family)	41	31.8 %
Degree of toy detail	Low level (a doctor bear)	19	14.7 %
	Middle level (a doctor doll)	28	21.7 %
	High level (a hospital play set)	82	63.6 %

### Socio-demographic factors and developmental characteristics impact on toy preferences

The socio-demographic factors that could affect children's toy preferences included in this study were their age and gender, the mother’s level of education, and the number of siblings. Gender-related specifics of toy preferences and play behavior were explored by using the Independent Samples T-Test. The analysis revealed significant differences between girls and boys only in the degree of toy detail trial (t( 127) = 2.36, p = 0.020). In that trial, the boys chose more detailed toys significantly more often than the girls (M = 2.64, SD = 0.67; M = 2.34, SD = 0.77, respectively). No significant gender-related differences were found in the trials on the degrees of realism and anthropomorphism.

Correlation analysis was applied to explore the potential relationship of toy preferences and the children's ages, their mothers’ education level, and their number of siblings. The only variables that demonstrated significant correlation were the number of siblings and the degree of realism of the toy (r = .278, p = 0.01). The more siblings a child had, the more he or she tended to choose the most realistic toys compared to toys not related to real experiences.

Next, correlation analysis was used to answer the question of whether developmental characteristics significantly predict a child’s toy preferences. The number of siblings in the family was taken as the control variable because this factor turned out to be significantly correlated to some toy preferences. However, no significant associations were found between the toy preferences and children’s non-verbal intelligence, executive function skills, or emotional understanding (p > 0.05).

## Discussion

As mentioned in the Introduction, the currently common toy typologies and categories are mainly decided by manufacturers and retailers of children’s products. Thus, toys often are categorized according to the technology by which they are constructed. This approach does not provide valuable information about what opportunities a particular toy provides for a child’s play and development, because it is not based on a theoretical understanding of developmental principles (Veraksa et al., 2022).

This study proposed three criteria for categorizing toys based on the cultural-historical approach: the degree of toy realism (Vygotsky, 1972); the degree of toy anthropomorphism (Elkonin, 2005); and the degree of toy detail (Smirnova, 2011). In the cultural-historical approach, play is understood as a source of child development. In play, the child recreates the events and processes of real life and thereby learns how to deal with them. In play, the child achieves a better understanding of the world and the culture he or she is growing up in. Realistic, anthropomorphic, and detailed toys are supposed to have a special value for play because they help to create an imaginative play situation that is closest to reality.

A toy preference experiment was conducted on a sample of 129 3-to-4-year-old children to test the performance of the proposed toy categorization criteria. The data from the children’s choice of toys in the three experimental trials supported the assumption that children would prefer more realistic and detailed toys. However, in the test for the degree of anthropomorphism of the toy, the expected result was not obtained. Children were expected to be more likely to choose a human family. But the most frequent choice in this sample was a family of pigs that look like humans. This result is probably due to the popularity of the character from the Peppa Pig™ play set. Children may have chosen the pig family because they were familiar with the brand *(e.g.,* from watching cartoons). This risk was assumed in the design of the study. However, no alternative option was found in the toy market (see Limitations).

The present study also analyzed the impact of socio-demographic factors (gender and age of the children, the level of the mothers education, and the number of siblings) on toy preferences. We found that the number of siblings was a statistically significant predictor of children's preferences for more realistic toys. The more children there were in the family, the more often they chose more realistic toys over those that were unrelated to their life experiences. It was also found that boys tended to choose more detailed toys than girls. The lack of correlation between play and the age of children can be interpreted as a confirmation of the universality of the play need at an early age, which actively develops regardless of the socio-demographic factors determining the children's environment.

For the first time, this study also considered a child’s developmental characteristics (non-verbal intelligence, executive functions, and emotional understanding) as possible predictors of preference for particular toys. Still, this experiment did not detect any evidence for such a relationship.

## Conclusion

This study proposed three criteria for categorizing toys based on the cultural-historical approach: the degree of realism, the degree of anthropomorphism, and the degree of detail of the toy. These criteria were highlighted as a result of analysis of theoretical works carried out in the framework of cultural-historical approach. It was assumed that realistic, anthropomorphic, and detailed toys have a special value for play because they help the child to create lifelike play situations and explore the world through them. The proposed criteria were tested through an experiment on the children's toy preferences. Experimental data confirmed that most children do prefer realistic and detailed toys to those with fewer of these properties. It was revealed that among various socio-demographic factors, only the child’s gender and the number of siblings in the family was significant predictors for the toy preferences. None of child’s developmental characteristics (non-verbal intelligence, executive functions, and emotional understanding) were found to be significant predictors of preference for particular toys.

## Limitations

Among the limitations of this research one can include the narrow age coverage of the sample that only included 3-to-4-year-old children, as well as certain flaws related to the application of the forced choice paradigm to explore the participants’ toy preferences. Compared to the naturalistic approach, or free play analysis, this paradigm implies the formation of toy sets to be offered under certain criteria, together with the experimenter’s presence while the child makes his/her choice. Both these factors can potentially affect the expressed preference. However, the naturalistic approach (the analysis of the toys that belong to the child) would entail even more limitations. For instance, these toys do not always reflect real interests and affections of children, but rather the values and the preferences of the adults that purchased them. There are concerns that in the experimental anthropomorphic trial, the toys were not selected optimally. In this trial children chose toys with a medium level of anthropomorphism (the pig family), which was probably due to the fact that children recognized their similarities with popular animated heroes (Peppa Pig™ play set.).
